# Risk factors for revascularization and in-stent restenosis in patients with triple-vessel disease after second-generation drug-eluting stent implantation: a retrospective analysis

**DOI:** 10.1186/s12872-021-02259-0

**Published:** 2021-09-17

**Authors:** MengYing Zeng, XiaoWei Yan, Wei Wu

**Affiliations:** Department of Cardiology, Peking Union Medical College Hospital (Dongdan Campus), Chinese Academy of Medical Sciences & Peking Union Medical College, No. 1 Shuaifuyuan Wangfujing Dongcheng District, Beijing, 100730 China

**Keywords:** Triple-vessel coronary artery disease, Revascularization, In-stent restenosis, Second-generation drug-eluting stents, Risk factors

## Abstract

**Objectives:**

Coronary artery disease (CAD) is a common cardiac disease with high morbidity and mortality, and triple-vessel disease (TVD) is a severe type of CAD. This study investigated risk factors for revascularization and in-stent restenosis (ISR) in TVD patients who underwent second-generation drug-eluting stent implantation.

**Methods:**

A retrospective clinical study was conducted, and 246 triple-vessel disease (TVD) patients with 373 vessels after second-generation drug-eluting stent (DES) implantation who received follow-up coronary angiography (CAG) were consequently enrolled. According to the follow-up angiography, patients were categorized into the revascularization group and nonrevascularization group as well as the in-stent restenosis (ISR) group and non-ISR group. Univariate and multivariate logistic regression analyses were used to identify risk factors for revascularization and ISR. Receiver operating characteristic (ROC) curve with area under the curve (AUC) analysis was performed to assess the predictive power of these risk factors.

**Results:**

In the median follow-up period of 28.0 (14.0, 56.0) months, 142 TVD patients (57.7%) with 168 vessels underwent revascularization, and ISR occurred in 43 TVD patients (17.5%) with 47 vessels after second-generation DES implantation. Compared to the nonrevascularization group, the revascularization group presented with an increased rate of current smoking and higher levels of TC, LDL-C, HDL-C, non-HDL-c, ApoB, neutrophils, and Hs-CRP as well as a longer follow-up of months but with a lower level of HDL-C. In addition, patients in the ISR group had an older age, longer follow-up (months) and elevated rates of current smoking and stage 4–5 chronic kidney disease (CKD4-5). In multivariate analysis, current smoking and higher non-HDL-c were independent risk factors for revascularization. In addition, older age, current smoking and CKD4-5 were considered independent risk factors for ISR. Importantly, the receiver operating characteristic curve showed that non-HDL-C and age displayed predictive power for revascularization and ISR, respectively.

**Conclusion:**

Current smoking is an independent risk factor for both revascularization and in-stent restenosis. Higher non-HDL-c is independently related to revascularization; moreover, increased age and CKD4-5 are potential risk factors for ISR in TVD patients after second-generation drug-eluting stent implantation.

## Introduction

Coronary artery disease (CAD) is a leading cause of death worldwide. Triple-vessel disease (TVD) is defined as ≥ 50% narrowing in all three major epicardial coronary arteries (left anterior descending artery, LAD; left circumflex artery, LCX; right coronary artery, RCA) with or without left main coronary artery disease (LM), which is a severe type of CAD. Additionally, TVD is regarded as an independent predictor of major adverse cardiac events (MACEs) and all-cause mortality [1, 2]. Over the past two decades, percutaneous coronary intervention (PCI) has become a primary modality for coronary revascularization, even for TVD patients. Drug-eluting stents (DESs) in patients with CAD (acute coronary syndrome or stable angina) can improve clinical outcomes when compared to bare-metal stents [3] but the occurrence rate of angiographic stenotic progression, such as revascularization and in-stent restenosis, remains high in CAD patients, especially for late or very late restenosis [4]. Some studies have explored ISR risk or risk factors for revascularization in CAD patients after PCI, indicating that dyslipidemia, diabetes mellitus, hypersensitive C-reactive protein, smoking and homocysteine, vessel size and complex lesion morphology were closely associated with ISR and revascularization [5–8]. However, little is known about the risk factors for revascularization and ISR in TVD patients after second-generation DES implantation. Therefore, we conducted this study to investigate the risk factors in these patients.

## Materials and methods

### Study patients

Patients with TVD after second-generation DES implantation who received follow-up CAG at the Department of Cardiology, Peking Union Medical College Hospital between February 2015 and November 2020 were consecutively enrolled in this retrospective study. The inclusion criteria were as follows: (a) patients diagnosed with TVD and at least one of the 3 major coronary arteries underwent second-generation DES implantation, (b) age older than18 years old, (c) patients receiving follow-up CAG after the previous procedure, and (d) patients receiving dual antiplatelet therapy (DAPT). The exclusion criteria were as follows: (a) severe liver dysfunction disease; (b) combined myocarditis, congenital heart disease, valvular diseases, cardiomyopathy, autoimmune disease, malignancies, infectious diseases and hyperthyroidism or hypothyroidism; (c) contraindications to aspirin, clopidogrel or ticagrelor; (d) follow-up CAG was not available; and (e) discontinuing antiplatelet therapy without medical advice. This study was approved by the Ethics Committee of Peking Union Medical College Hospital and performed in accordance with Declaration of Helsinki. Written informed consent was obtained from all patients.

### Data collection

Baseline parameters, including demographic information, risk factors related to CAD (hypertension, diabetes mellitus, family history of CAD, CKD, current smoking and alcohol intake), laboratory tests (lipid profile, in which, non-HDL-c was calculated by subtracting the HDL-c level from the TC level, neutrophils, lymphocytes, white blood cells [WBCs], hypersensitive C-reactive protein [hs-CRP], glycosylated hemoglobin A1c [HbA1c] and homocysteine, serum uric acid [SUA]), were collected before the follow-up CAG. The follow-up time and CAG findings were also included. Second generation DES were used in our patients, including sirolimus-eluting stents (Microport, Shanghai, China and Jiwei, Shandong, China), everolimus-eluting stents (Boston Scientific, Natick, Massachusetts, and Abbott Vascular, Santa Clara, California), and zotarolimus-eluting stents (Medtronic, Santa Rosa, California). To our knowledge, everolimus-eluting stents and zotarolimus-eluting stents are used in current clinical practice worldwide.

### PCI and grouping

After previous stent implantation, patients received 100 mg aspirin and 75 mg clopidogrel once daily or 90 mg ticagrelor twice daily. Lesion progress and ISR in the follow-up CAG were evaluated by two independent interventional cardiologists. ISR was defined as percent diameter stenosis ≥ 50% in the stent at follow-up angiography. Revascularization was defined as receiving second revascularization in the same lesions or different lesions at follow-up angiography. Patients who required revascularization in follow-up were included in the revascularization group. Patients with ISR were included in the ISR group.

### Statistical analysis

All statistical analyses were performed using SPSS (version 23), and continuous variables were reported as the mean ± standard deviation (X ± SD) or median (interquartile range) according to whether they were normally distributed. Categorical variables were expressed as frequencies [n, (%)]. Intergroup measurement comparisons were performed using t-tests or Wilcoxon rank sum tests, and counts were compared by chi-square tests. Univariate logistic regression analysis and multivariate logistic regression model analysis were used to determine risk factors for revascularization and ISR with odds ratios (ORs) and 95% confidence intervals (CIs). Receiver operating characteristic (ROC) curve with area under the curve (AUC) analysis was performed to assess the predictive power of risk factors for revascularization and ISR. All *P* values were two-sided, and *P* < 0.05 was considered statistically significant.

## Results

### TVD patient characteristics

**Fig. 1:**
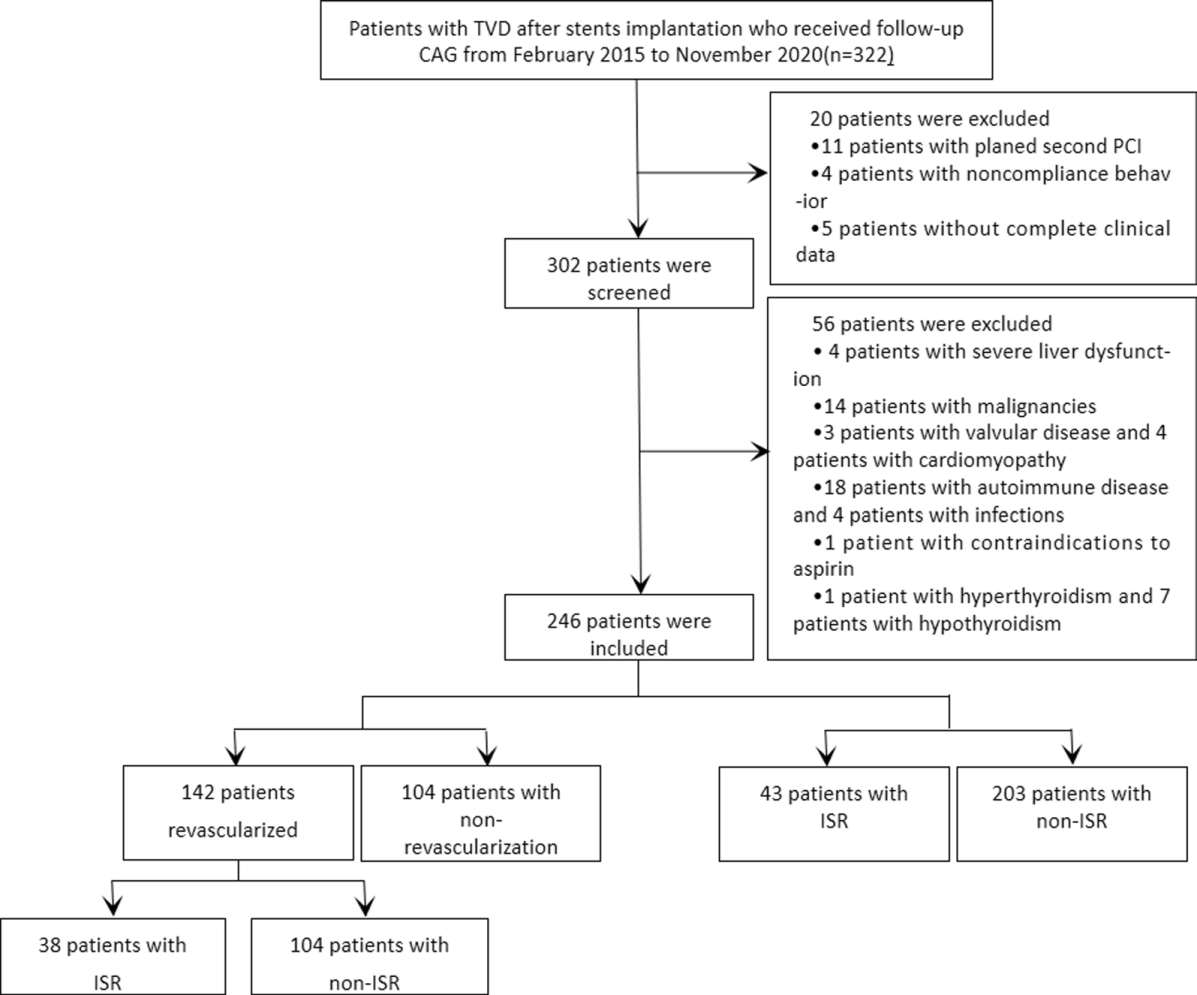
Study flow

Over a median follow-up of 28 (14.0, 56.0) months, the study enrolled 246 TVD patients (373 vessels) who underwent follow-up CAG after second-generation DES stent implantation (Fig. 1), of whom 70.7% were men. The mean age was 64.3 ± 10.0 years. A total of 202 (82.1%) patients received follow-up CAG due to angina pectoris or precordial distress. In contrast, 44 (17.9%) patients received routine follow-up CAG. According to the laboratory results, the patients’ mean non-HDL-C was 2.63 ± 0.81 mmol/L, and the mean LDL-C was 1.95 ± 0.63 mmol/L. In the previous PCI procedure, 37.8% of patients received two-vessel stenting, and 6.9% of patients underwent triple-vessel stenting. Other characteristics of TVD patients are shown in Table 1.

**Table 1:** Population characteristics of TVD patients who received follow-up CAG after second-generation DES implantation

Population characteristics	TVD patients (n = 246)	Revascularization group (n = 142)	Nonrevascularization group (n = 104)	*P* value	ISR group (n = 43)	Non-ISR group (n = 203)	*p* value
Age (years)	64.3 ± 10.0	63.8 ± 9.9	65.0 ± 10.0	0.340	67.3 ± 10.2	63.6 ± 9.8	0.029•
Male (n, %)	174 (70.7%)	101 (71.1%)	73 (70.2%)	0.874	31 (72.1%)	143 (70.4%)	0.829
BMI (kg/m^2^)	26.2 ± 3.3	26.2 ± 3.4	26.2 ± 3.2	0.948	26.1 ± 2.9	26.2 ± 3.3	0.858
Current smoking (n, %)	92 (37.4%)	66 (46.5%)	26 (25.0%)	0.001*	22 (51.2%)	70 (34.5%)	0.040•
Current alcohol intake (n, %)	66 (26.8%)	41 (28.9%)	25 (24.0%)	0.398	10 (23.3%)	56 (27.6%)	0.560
Hypertension (n, %)	186 (75.6%)	112 (78.9%)	74 (71.2%)	0.164	32 (74.4%)	154 (75.9%)	0.841
SBP > 140 and/or DBP > 90 (n, %)	70 (29.3%)	38 (26.8%)	34 (32.4%)	0.312	10 (23.3%)	62 (30.5%)	0.340
Diabetes (n, %)	129 (52.4%)	81 (57.0%)	48 (46.2%)	0.091	24 (55.8%)	105 (51.7%)	0.626
Family history of CAD (n, %)	76 (30.9%)	45 (31.7%)	31 (29.8%)	0.752	12 (27.9%)	64 (31.5%)	0.641
CKD1-3 (n, %)	20 (8.1%)	13 (9.2%)	7 (6.7%)	0.492	5 (11.6%)	15 (7.4%)	0.356
CKD4-5 (n, %)	9 (3.7%)	9 (6.3%)	0	/	4 (9.3%)	5 (2.5%)	0.030•
Angina pectoris or precordial distress (n, %)	202 (82.1%)	136 (95.8%)	66 (63.5%)	< 0.001*	38 (88.4%)	164 (80.8%)	0.238
*Laboratory test*							
TC (mmol/L)	3.63 ± 0.84	3.76 ± 0.90	3.44 ± 0.74	0.005*	3.84 ± 1.04	3.58 ± 0.79	0.073
LDL-C (mmol/L)	1.95 ± 0.63	2.07 ± 0.69	1.79 ± 0.51	0.001*	2.00 ± 0.75	1.94 ± 0.61	0.552
HDL-C (mmol/L)	1.00 ± 0.24	0.97 ± 0.22	1.05 ± 0.26	0.004*	1.01 ± 0.30	0.99 ± 0.23	0.674
Non-HDL-C (mmol/L)	2.63 ± 0.81	2.80 ± 0.75	2.40 ± 0.66	< 0.001*	2.83 ± 1.05	2.59 ± 0.75	0.083
ApoB (g/L)	0.74 ± 0.20	0.77 ± 0.20	0.70 ± 0.18	0.009*	0.74 ± 0.19	0.74 ± 0.20	0.788
NEU (× 10^9^)	4.39 ± 1.40	4.59 ± 1.48	4.11 ± 1.23	0.008*	4.72 ± 1.46	4.31 ± 1.38	0.078
LY (× 10^9^)	1.82 ± 0.69	1.80 ± 0.66	1.82 ± 0.73	0.874	1.80 ± 0.65	1.81 ± 0.70	0.923
NLR	2.40 (1.88, 3.08)	2.43 (1.91, 3.29)	2.26 (1.81, 2.86)	0.120	2.50 (2.11, 3.58)	2.39 (1.86, 3.05)	0.179
WBC (× 10^9^)	6.82 ± 1.82	7.04 ± 1.83	6.52 ± 1.79	0.672	7.15 ± 1.95	6.74 ± 1.80	0.184
Hs-CRP (mg/L)	0.96 (0.43, 2.22)	1.22 (0.53, 2.68)	0.83 (0.39, 1.88)	0.022*	1.38 (0.70, 3.23)	0.92 (0.40, 2.15)	0.066
HbA1c (%)	6.3 (5.8, 7.3)	6.4 (5.8, 7.8)	6.2 (5.8, 6.8)	0.090	6.4 (6.0, 8.4)	6.2 (5.8, 7.1)	0.050
SUA (μmol/L)	351 (296, 403)	357(297, 409)	339 ± 78	0.320	341 (289, 401)	354 (299, 405)	0.680
*Previous findings*							
ACS (n/%)	154 (62.6%)	88 (62.0%)	66 (63.5)	0.811	19 (44.2%)	73 (36.0%)	0.311
Target vessel at LM (n/%)	22 (8.9%)	9 (5.3%)	13 (12.5%)	0.094	2 (4.7%)	20 (9.9%)	0.278
Target vessel at LAD only (n/%)	63 (25.6%)	36 (25.4%)	27 (26.0%)	0.914	14 (32.6%)	49 (24.1%)	0.250
Target vessel at LCX only (n/%)	29 (11.8%)	18 (12.7%)	11(10.6%)	0.614	0 (0%)	29 (4.3%)	/
Target vessel at RCA only (n/%)	45 (18.3%)	26 (18.3%)	19 (18.3%)	0.994	6 (14.0%)	39 (19.2%)	0.418
Two vessels stenting (n/%)	93 (37.8%)	53 (37.3%)	40 (38.5%)	0.856	18 (41.9%)	75 (36.9%)	0.546
Triple vessels stenting (n/%)	17 (6.9%)	10 (7.0%)	7 (6.7%)	0.924	5 (11.6%)	12 (5.9%)	0.179
SYNTAX score	12.9 ± 4.5	13.4 ± 4.7	12.4 ± 4.0	0.085	12.6 ± 5.3	13.0 ± 4.3	0.558
Everolimus/zotarolimus-eluting stents (n/%)	184 (74.8%)	107 (75.4%)	77 (74.0%)	0.815	31 (72.3%)	153 (75.4%)	0.653
Time of follow-up (months)	28.0 (14.0,56.0)	16.8 (38.0,63.0)	23.5 (13.0,48.5)	0.002*	39.0 (22.0,78.0)	27.0 (14.0, 53.0)	0.024•

Data are presented as the mean ± SD, median (interquartile range) and (n/%)

*TVD* triple-vessel disease, *CAG* coronary angiography, *DES* drug-eluting stents, *ISR* in-stent restenosis, *BMI* body mass index, *SBP* systolic blood pressure, *DBP* diastolic blood pressure, *CAD* coronary artery disease, *CKD* chronic kidney disease, *TC* total cholesterol, *LDL-C* low-density lipoprotein cholesterol, *HDL-C* high-density lipoprotein cholesterol, *non-HDL-C* non-high-density lipoprotein cholesterol, *ApoB* apolipoprotein B, *NEU* neutrophil, *LY* lymphocyte, *NLR* neutrophil-to-lymphocyte ratio, *WBC* white blood cell, *Hs-CRP* high-sensitivity C-reactive protein, *HbA1c* glycosylated hemoglobin A1c, *HCY* homocysteine, *SUA* serum uric acid, *LM* left main coronary artery disease, *LAD* left anterior descending branch, *LCX* left circumflex artery RCA, right coronary artery

*P < 0.05, compared with nonrevascularization cases; •*P* < 0.05, compared with non-ISR cases

### Comparison of characteristics between revascularization group and nonrevascularization group as well as ISR group and non-ISR group

A total of 142 patients (168 vessels) required revascularization according to the follow-up CAG, among which 4 patients were recommended to undergo coronary artery bypass graft (CABG) surgery. Compared to the nonrevascularization group, more patients in the revascularization group were current smokers (46.5% versus 25.0%, *P* = 0.001). The revascularization group had lower HDL-c levels (0.97 ± 0.22 mmol/L versus 1.05 ± 0.26 mmol/L, *P* = 0.004) but higher non-HDL-c (2.80 ± 0.75 mmol/L versus 2.40 ± 0.66 mmol/L, *P* < 0.001), TC (3.76 ± 0.90 mmol/L versus 3.44 ± 0.74 mmol/L, *P* = 0.005), LDL-C (2.07 ± 0.69 mmol/L versus 1.79 ± 0.51 mmol/L, *P* = 0.001) and ApoB (0.77 ± 0.20 mmol/L versus 0.70 ± 0.18 mmol/L, *P* = 0.009) levels than the nonrevascularization group. Moreover, in the revascularization group, patients had higher counts of neutrophils (P = 0.008) and hs-CRP (*P* = 0.022) as well as more months of follow-up (*P* = 0.002). In total, 43 patients (47 vessels) were confirmed as ISR. Compared to the non-ISR group, patients were older in the ISR group (67.3 ± 10.2 versus 63.6 ± 9.8, *P* = 0.029). Furthermore, ISR patients had a higher percentage of current smokers (*P* = 0.040) and CKD4-5 (*P* = 0.030) than non-ISR patients. In addition, the follow-up time was longer in the ISR group compared with the non-ISR group (*P* = 0.024). (Table 1).

### Revascularization and ISR rates at different follow-up times

**Fig. 2:**
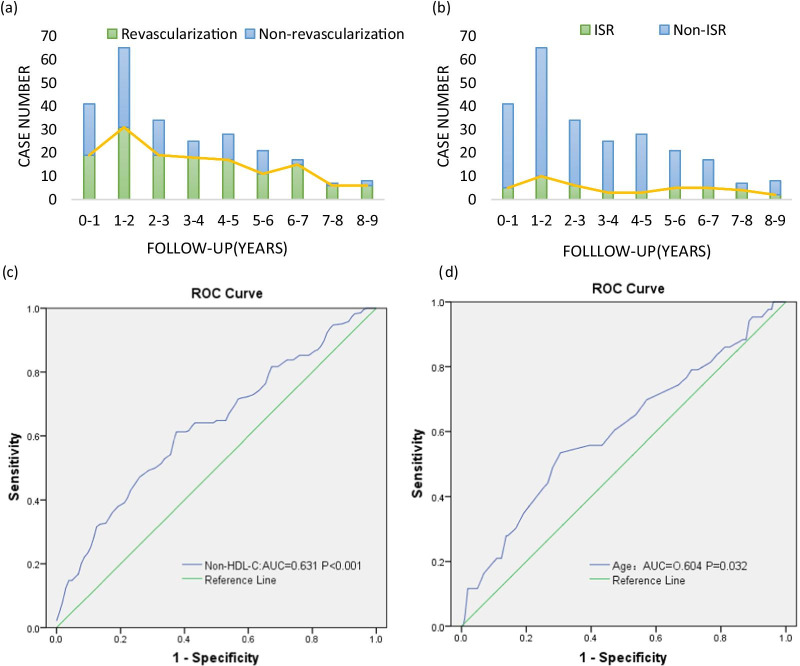
**a** The number of cases of revascularization and nonrevascularization based on the follow-up duration in years; **b** The number of cases of in-stent restenosis and non-in-stent restenosis based on the follow-up duration in years. **c** Receiver operating characteristic (ROC) curve analysis of non-HDL-C for predicting revascularization; **d** Receiver operating characteristic (ROC) curve analysis of ages for predicting in-stent restenosis

As illustrated in Fig. 2a and b, the percentages of revascularization and ISR were both the highest in the 1st to 2nd year after stent implantation with values of 12.6% (31 of 246 patients) and 4.1% (10 of 246 patients), respectively. On the one hand, the incidence of revascularization reached the second peak in the 6th to 7th follow-up years. On the other hand, the rate of ISR gradually decreased with the prolongation of the follow-up time until the 5th to 6th year.

### Logistic regression analysis of risk factors for revascularization

Univariate logistic regression analysis revealed that current smoking (*P* = 0.001, OR = 2.605, 95% CI 1.499–4.529) was positively correlated with the risk of revascularization. We confirmed that TC (*P* = 0.006, OR = 1.591, 95% CI 1.415–2.111), LDL-C (*P* = 0.001, OR = 2.153, 95% CI 1.354–3.423), non-HDL-c (*P* < 0.001, OR = 1.994, 95% CI 1.378–2.887), ApoB (*P* = 0.010, OR = 7.549, 95% CI 1.610–35.405), NEU (*P* = 0.010, OR = 1.301, 95% CI 1.066–1.588) and follow-up time (*P* = 0.002, OR = 1.017, 95% CI 1.006–1.027) were positively related to revascularization risk. In contrast, HDL-c (*P* = 0.011, OR = 0.2248, 95% CI 0.084–0.730) was negatively correlated with revascularization risk. After adjusting for all the possible risk factors, multivariate logistic regression analyses showed that current smoking (*P* < 0.001, OR = 3.238, 95% CI 1.716–6.112) and non-HDL-c (*P* = 0.001, OR = 1.983, 95% CI 1.325–2.969) were independent risk factors for follow-up revascularization in TVD patients who underwent second-generation DES implantation (Table 2).

**Table 2:** Univariate and multivariate logistic regression analysis for risk factors related to revascularization in TVD patients after second generation DES implantation

Factors	Univariate analysis			Multivariate analysis		
	OR	95% CI	*P* value	OR	95% CI	*P* value
Current smoker	2.605	1.499–4.529	0.001	3.238	1.716–6.112	< 0.001
TC	1.591	1.145–2.211	0.006	–	–	NS
LDL-C	2.153	1.354–3.423	0.001	–	–	NS
HDL-C	0.214	0.072–0.632	0.005	–	–	NS
Non-HDL-C	1.994	1.378–2.887	< 0.001	1.983	1.325–2.969	0.001
ApoB	7.549	1.610–35.405	0.010	–	–	NS
NEU	1.301	1.066–1.588	0.010	–	–	NS
Follow-up time	1.017	1.006–1.027	0.002	–	–	NS

*TVD* triple-vessel disease, *DES* drug-eluting stents, *CKD* chronic kidney disease, *TC* total cholesterol, *LDL-C* low-density lipoprotein cholesterol, *HDL-C* high-density lipoprotein cholesterol, *non-HDL-C* non-high-density lipoprotein cholesterol, *ApoB* apolipoprotein B, *NEU* neutrophil, *OR* odds ratio, *CI* confidence interval, *NS* no significant association (*P* > 0.05)

### Logistic regression analysis of risk factors for ISR

Based on univariate logistic regression analysis, the risk factors for ISR included age (*P* = 0.030, OR = 1.040, 95% CI 1.004–1.077), current smoking (*P* = 0.042, OR = 1.990, 95% CI 1.024–3.868), CKD4-5 (*P* = 0.043, OR = 4.062, 95% CI 1.044–15.807) and follow-up time (*P* = 0.012, OR = 1.015, 95% CI 1.003–1.027). In multivariate logistic regression analyses after adjusting for all the possible risk factors, older age, current smoking and CKD4-5 were independent factors for predicting an increased occurrence of ISR (*P* = 0.004, OR = 1.060, 95% CI 1.019–1.102, *P* = 0.006, OR = 2.918, 95% CI 1.367–6.227 and *P* = 0.042, OR = 4.985, 95% CI 1.056–23.533, respectively) (Table 3).

**Table 3:** Univariate and multivariate logistic regression analysis of risk factors related to revascularization in TVD patients after second-generation DES implantation

Factors	Univariate analysis			Multivariate analysis		
	OR	95% CI	*P* value	OR	95% CI	*P* value
Age	1.040	1.004–1.077	0.030	1.060	1.019–1.102	0.004
Current smoking	1.990	1.024–3.868	0.042	2.918	1.367–6.227	0.006
CKD4-5	4.062	1.044–15.807	0.043	4.985	1.056–23.533	0.042
Follow-up time	1.015	1.003–1.027	0.012	–	–	NS

*TVD* triple-vessel disease, *DES* drug-eluting stents, *HbA1c* glycosylated hemoglobin A1c, *OR* odds ratio, *CI* confidence interval, *NS* no significant association (*P* > 0.05)

### Receiver operating characteristic (ROC) curve analysis of risk factors for predicting revascularization and ISR

ROC curve analysis demonstrated that a non-HDL-C value of 2.52 mmol/L was the cutoff level based on the Youden index analysis, with a sensitivity and specificity of 60.6% and 63.5% (AUC = 0.631, 95% CI = 0.562–0.700, *P* < 0.001) (Fig. 2c). In the ROC curve analysis of age (AUC = 0.604, 95% CI = 0.506–0.702, *P* = 0.032), 67.5 years of age was determined to be a predictive cutoff point for ISR, with a sensitivity and specificity of 53.5% and 77.1%, respectively (Fig. 2d).

## Discussion

This retrospective study revealed several findings. First, the total incidence rate of revascularization was 57.7%, and the rate of ISR was 17.5% in the long-term follow-up study. Second, late adverse events, such as late ISR and late revascularization, even very late ISR and revascularization, continue to occur beyond 1 year after second-generation DES implantation. Third, current smoking was an independent risk factor for both revascularization and in-stent restenosis. Higher non-HDL-c is independently related to revascularization, and older age and CKD4-5 are potential risk factors for ISR in TVD patients after second-generation DES implantation. Moreover, non-HDL-C and age displayed predictive power in revascularization and ISR, respectively.

Several randomized controlled trials demonstrated sustained benefit of DES without major safety concerns compared to BMS [9, 10]. However, adverse events after DES implantation, such as revascularization and ISR, remain an important clinical problem. A prospective study indicated that any revascularization occurred in 16.5% of CAD patients at 6 years [11]. Another registered study showed that the cumulative incidence of any revascularization in CAD patients was 38.6% at 5 years [12]. In our study, the cumulative rate (57.7%) of revascularization was greater than that in the abovementioned studies. The possible reasons were as follows: First, all individuals included in the study were TVD patients, and these patients are classified as high-risk CAD patients. Second, the follow-up time was longer than that in previous studies, and more risk factors may accumulate with the prolonged follow-up time. Third, 202 (82.1%) patients received follow-up CAG due to angina pectoris or precordial distress, which may cause the increased incidence of revascularization. The incidence of in-stent restenosis varied in different studies. One clinical study showed that at 2 years, the cumulative incidence of restenosis was 20% in CAD patients [13], and another study concluded that the incidence of restenosis in three-vessel disease was 20.9%, with a mean 45.6 ± 21.5 months [14]. In the present study, the rate of ISR was 17.5%, with a median of 28.0 (14.0, 56.0) months. Our study showed that the ISR data were comparable.

Studies have indicated an increase in the incidence of revascularization and ISR over time across different generations of DESs [15, 16]. Late and very late ISR continued to occur constantly without attenuation up to 5 years after DES implantation [12]. Our present study demonstrated that angiographic stenotic progression (revascularization and ISR) was a continuous hazard, and a late catch-up phase occurs at 5 + years after second-generation DES implantation. After stent implantation, fibrin deposition substitution for smooth muscle cells was the main process of neointima healing, and the best predictor of neointima was a 20-month follow-up period after drug stent implantation [17]. Progressive neointima may lead to neoatherosclerosis with a median stent duration of 420 days and contribute to angiographic stenotic progression [18]. It is worth noting that the trend of revascularization and ISR rates after DES implantation in our study can be explained by the abovementioned mechanism.

Plaque rupture and subsequent injury response facilitate the accretion of the vascular wall, contributing to angiographic stenotic progression. Patients who smoke after PCI have more rupture-prone unstable plaques and angina than patients who do not smoke [19]. Therefore, smoking may cause angiographic stenotic progression in patients. Although published studies have reported conflicting results about the relationship between smoking and revascularization or ISR in CAD patients after stent implantation [20–22], our results suggested that smoking after DES implantation served as a risk factor for revascularization and ISR (OR = 1.990, 95% CI 1.024–1.077 and OR = 2.717, 95% CI 1.268–5.821, respectively) in TVD patients.

Non-HDL-c was calculated by subtracting the HDL-c level from the TC level, and we demonstrated that non-HDL-c may be a potential predictor of risk for revascularization in TVD patients after second-generation DES stent implantation (AUC = 0.631, *P* < 0.001). Non-HDL-c encompasses not only LDL-C, intermediate density lipoprotein and lipoprotein (a) but also very low-density lipoprotein cholesterol (VLDL-c), which can aid in increased predictive power. Moreover, non-HDL-c was considered a surrogate for LDL particle number (LDL-P) assessed by either apoB or nuclear magnetic resonance (NMR) spectroscopy [23, 24]. Furthermore, non-HDL-c can be calculated in the nonfasting state or in the setting of hypertriglyceridemia, which is convenient for capturing lipid-associated risk prediction.

On the basis of the results, we verified older age as a predictor of ISR by logistic regression analysis and ROC curve analysis (AUC = 0.604, *P* = 0.032). Older age can independently predict the risk for ISR after stent implantation [25, 26]. Elderly patients with frequent and numerous comorbidities consistently exhibit decreased anticoagulant ability and thicker arterial walls, which makes them fragile with different phenotypes. Different phenotypes are differently associated with adverse events. What is more, elderly patients had a higher risk of being rehospitalized than younger patients, as well demonstrated in REPOSI study [27]. And these features contribute to the tendency to develop atherosclerosis and lead to restenosis. In addition, we observed that CKD4-5 was independently correlated with ISR risk. Patients with severe CKD or end-stage renal disease have a significantly higher risk of target lesion failure after second-generation DES implantation [28]. CKD is accompanied by high oxidative stress, endothelial dysfunction, and an inflammatory status and independently predicts neoatherosclerosis [29]. These factors can increase the risk of ISR in patients after DES implantation.

Diabetes mellitus (DM), which is generally considered an established risk factor for revascularization and in-stent restenosis after stent implantation [30], was not found to be an independent revascularization or ISR risk factor in our study. It has been reported that patients with DM and HbA1 < 7.0% undergoing stenting may benefit from reducing the risk of restenosis and experience lower rates of repeat revascularization [31]. And it was observed that intense glycemic control can improve the cardiovascular outcome after acute coronary syndrome even in non-diabetic hyperglycemic subjects [32, 33]. We believe that the low HbA1c level in our patients [median HbA1c 6.4% (5.8, 7.8) in the revascularization group and the median HbA1c 6.4% (6.0, 8.4) in the ISR group might be an important reason.

Accessing site crossover have been also associated with an increased risk of procedural failure and revascularization [34, 35]. The previous procedure was started via transradial approach in most of patients in our study. Few patients with complex lesions, such as chronic total occlusion received crossover (from transradial approach to transfemoral approach), some of them were recommended to undergo CABG surgery according to the result of CAG, which can contribute to reduce risk of procedural failure and revascularization.

## Limitations

This retrospective study still had some limitations. First, there was a lack of randomization, and the included patients were from a single center. Second, the number of patients included was relatively small, especially the sample of patients with ISR, and numerous of risk factors were included in the analysis, which might reduce the statistical power. Moreover, the correlation of other oral drugs that are related to CAD with revascularization and ISR was not investigated. Finally, the results of this study cannot be generalized to a younger population.

## Conclusion

In summary, our study demonstrated that a high risk of revascularization and ISR continues to exist in TVD patients after second-generation DES implantation, and angiographic stenotic progression (revascularization and ISR) is the continuous hazard. In addition, current smoking was an independent risk factor for both revascularization and in-stent restenosis. Higher non-HDL-c is independently related to revascularization. Moreover, older age and CKD4-5 are potential risk factors for ISR in TVD patients after second-generation drug-eluting stent implantation. For these patients, intense management of changes in lifestyle and better medical measures are needed to control risk factors. More clinical trials are needed to focus on these patients to elucidate high-risk factors for revascularization and ISR and to improve progress.

## Data Availability

All data generated or analyzed during the study are in the article.

## Abbreviations

TVD: triple-vessel disease
ISR: in-stent restenosis
DES: drug-eluting stent
BMS: bare-metal stent
CKD: chronic kidney disease
HDL-c: high-density lipoprotein cholesterol
CAD: coronary artery disease
LAD: left anterior descending artery
LCX: left circumflex artery
RCA: right coronary artery
LM: left main coronary artery disease
MACEs: major adverse cardiac events
PCI: percutaneous coronary intervention
DAPT: dual antiplatelet therapy
CAG: coronary angiography
CABG: coronary artery bypass graft
OR: odds ratios
CI: confidence intervals
ROC: receiver operating characteristic
AUC: area under the curve
BMI: body mass index;
SBP: systolic blood pressure
DBP: diastolic blood pressure
TC: total cholesterol
LDL-C: low-density lipoprotein cholesterol
HDL-C: high-density lipoprotein cholesterol
ApoB: apolipoprotein B
NEU: neutrophil
LY: lymphocyte
NLR: neutrophil-to-lymphocyte ratio
WBC: white blood cell
Hs-CRP: high-sensitivity C-reactive protein
HbA1c: glycosylated hemoglobin A1c
HCY: homocysteine
SUA: serum uric acid
DM: diabetes mellitus
